# ICTV Virus Taxonomy Profile: *Closteroviridae*

**DOI:** 10.1099/jgv.0.001397

**Published:** 2020-03-05

**Authors:** Marc Fuchs, Moshe Bar-Joseph, Thierry Candresse, Hans J. Maree, Giovanni P. Martelli, Michael J. Melzer, Wulf Menzel, Angelantonio Minafra, Sead Sabanadzovic, ICTV Report Consortium

**Affiliations:** ^1^​ School of Integrative Plant Science, Cornell University, Geneva, NY 14456, USA; ^2^​ The S. Tolkowsky Laboratory, Bet Dagan 50250, Israel; ^3^​ UMR 1332 Biologie du Fruit et Pathologie, INRA, University of Bordeaux, 33882 Bordeaux, France; ^4^​ Department of Genetics, Stellenbosh University and Citrus Research International, Stellenbosh, Western Cape, South Africa; ^5^​ Department of Soil, Plant and Food Sciences, Università degli Studi di Bari Aldo Moro, Bari, Italy; ^6^​ Department of Plant and Environmental Protection Sciences, University of Hawai’i at Manoa, Honolulu, HI 96822, USA; ^7^​ Leibniz-Institute DSMZ – German Collection of Microorganisms and Cell Cultures, Braunschweig, Germany; ^8^​ Department of Biochemistry, Molecular Biology, Entomology and Plant Pathology, Mississippi State University, Mississippi, MS 39762, USA

**Keywords:** *Closteroviridae*, ICTV Report, Taxonomy

## Abstract

Viruses in the family *Closteroviridae* have a mono-, bi- or tripartite positive-sense RNA genome of 13–19 kb, and non-enveloped, filamentous particles 650–2200 nm long and 12 nm in diameter. They infect plants, mainly dicots, many of which are fruit crops. This is a summary of the ICTV Report on the family *Closteroviridae*, which is available at ictv.global/report/closteroviridae.

## VIRION

**Fig. 1.**
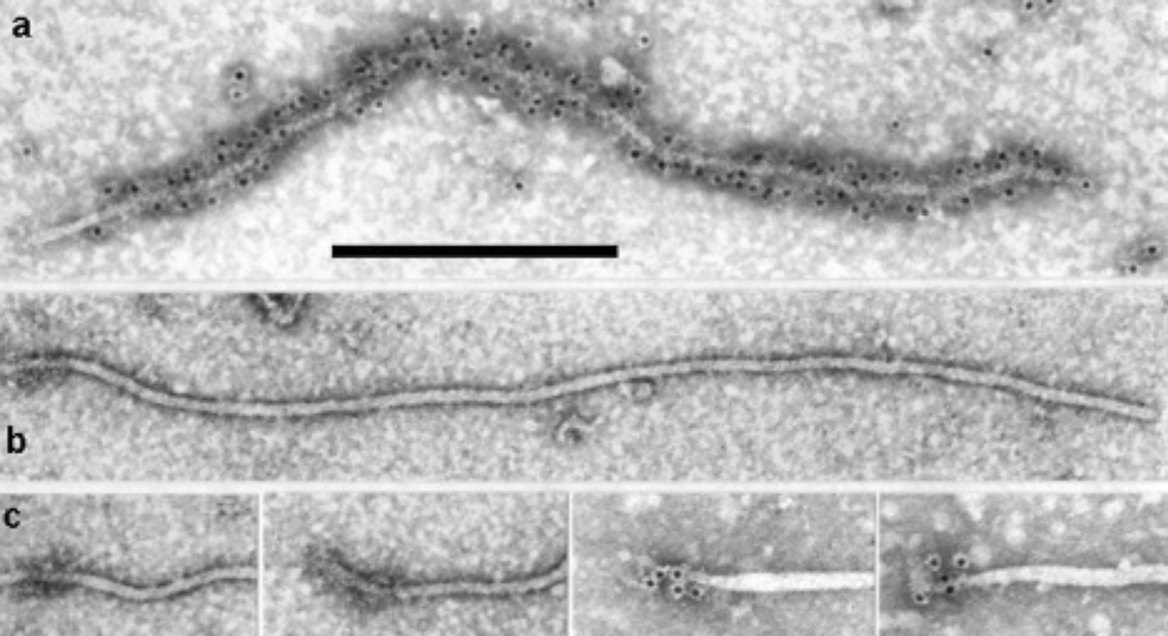
Electron micrographs of virions of beet yellows virus (genus *Closterovirus*) negatively-stained and decorated with an antiserum specific to (a) CP (bare for the CPm tail) and (b) CPm (75 nm tail only). (c) as (b) for four selected particles. Scale bar 300 nm. Reproduced with permission from [6].

Virions are long, helically constructed filamentous particles; the primary helix has a pitch of 3.4–3.8 nm, about 10 protein subunits per turn and a central hole of 3–4 nm (Table 1). The coat protein (CP) and minor CP (CPm) are the most abundant virion components. CPm encapsidates the 600–700 5′-terminal nucleotides of viral RNA (Fig. 1). The virus-encoded heat shock protein 70 homologue (HSP70h) and the ∼60 kDa protein are also integral to virions; a 20 kDa protein may form the tip of the virion head [1].

**Table 1.** Characteristics of members of the family *Closteroviridae*

Typical member:	citrus tristeza virus (U16304), species *Citrus tristeza virus*, genus *Closterovirus*
Virion	Non-enveloped, filamentous particles 650 to 2200 nm in length and 12 nm in diameter
Genome	13–19 kb of positive-sense, mono-, bi- or tripartite RNA
Replication	In association with endoplasmic reticulum-derived membranous vesicles and vesiculated mitochondria
Translation	Directly from genomic RNAs as large polyproteins or from sub-genomic mRNAs
Host range	Plants (mainly dicots), transmitted by aphids, whiteflies, mealybugs or soft-scale insects. No seed or pollen transmission
Taxonomy	Realm *Riboviria,* four genera, more than 50 species, some unassigned to a genus

## GENOME

**Fig. 2.**
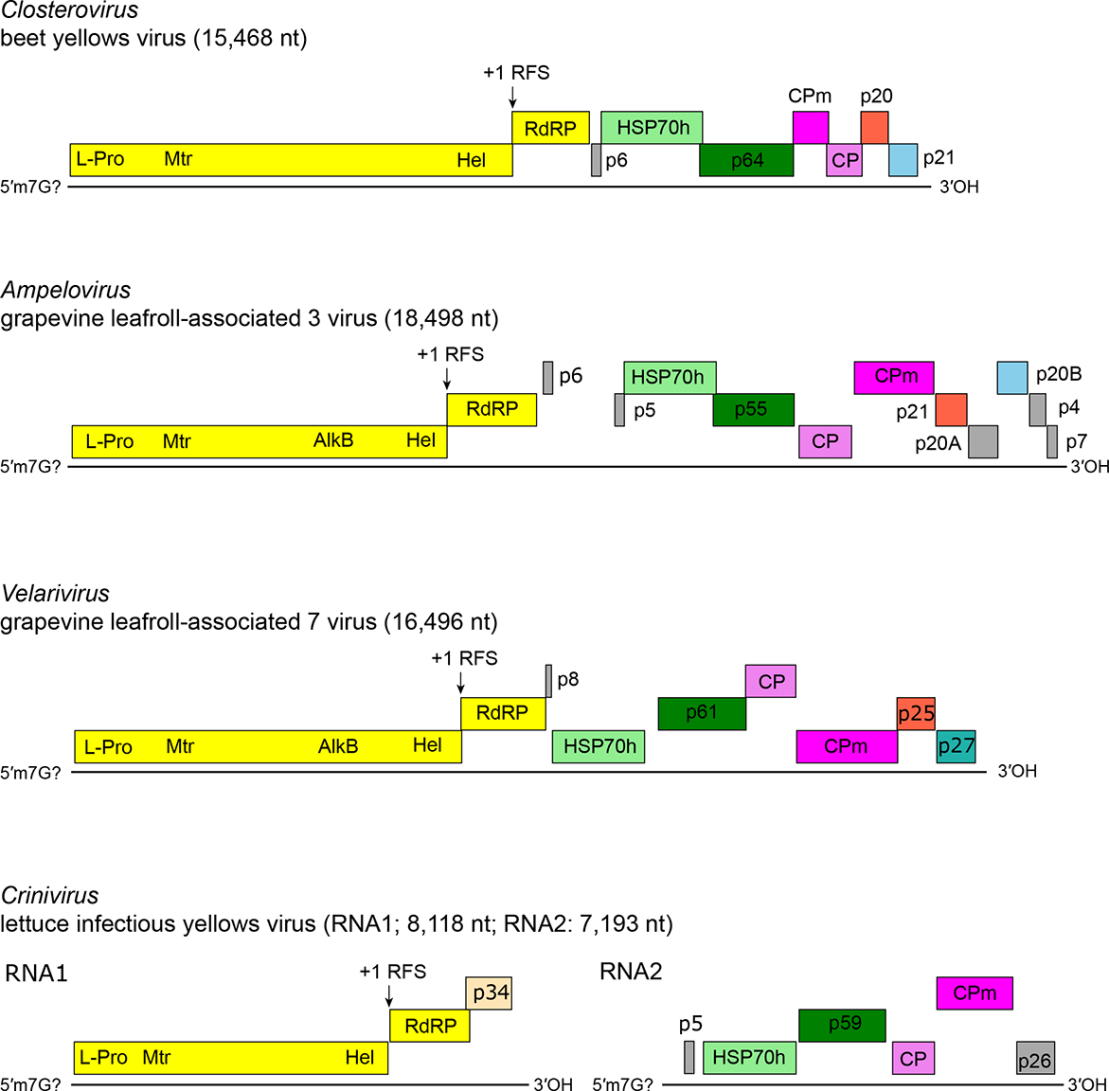
*Closteroviridae*: representative genome organisations.

The genome consists of 1–3 molecules of 5′-capped, linear, positive-sense RNA that lack a 3′-terminal poly(A) or tRNA-like structure (Fig. 2). The genome organization is conserved; the number and relative position of open reading frames (ORFs) can differ. The dual-gene module ORF1a–ORF1b at the 5′-end of genomic RNA encodes replication-associated proteins with conserved domains for a papain-like cysteine protease (l-Pro), methyltransferase (Met), helicase (Hel) and RNA-directed RNA polymerase (RdRP). Downstream ORFs form a conserved five-gene module encoding a 6K small hydrophobic protein, HSP70h, a ~60 kDa protein, CP and CPm [1]. Genome expression involves proteolytic processing of the polyprotein encoded by ORF1a; a +1 ribosomal frameshift for the expression of the RdRP domain of ORF1b; downstream ORFs expressed via nested 3′ co-terminal sub-genomic mRNAs (sgmRNAs) [2, 3].

## REPLICATION

Replication occurs in the cytoplasm, possibly in association with endoplasmic reticulum-derived membranous vesicles and vesiculated mitochondria induced by the 1a and 1b polyproteins [4]. Essential for replication are a conserved secondary structure at the 5′-untranslated region (UTR) and hairpin structures and a putative pseudoknot at the 3′-UTR of the genomic RNA. The transcription of sgmRNAs is temporally and quantitatively regulated, with each serving as a monocistronic messenger for translation of the corresponding 5′-proximal ORF.

## TAXONOMY

The genome of members of the genus *Closterovirus* is monopartite with CPm encoded upstream of CP. Transmission is by aphids in a semi-persistent manner [5]. Ampelovirus genomes are monopartite and show wide variation in size and organization. Transmission is by pseudococcid mealybugs and soft-scale insects in a semi-persistent manner. Crinivirus genomes are bi-or tripartite. Transmission is by whiteflies in a semi-persistent manner. Viruses in these three genera have a narrow host range and wide distribution; symptoms consist of foliar discoloration and deformation (yellowing, reddening, mottling, rolling), stunting and pitting. Velarivirus genomes are monopartite. Hemipteran vectors have not been identified; there are no apparent symptoms.

## RESOURCES

Current ICTV Report on the family *Closteroviridae*: ictv.global/report/closteroviridae.
